# CpG 1018 Is an Effective Adjuvant for Influenza Nucleoprotein

**DOI:** 10.3390/vaccines11030649

**Published:** 2023-03-14

**Authors:** Yibo Li, Xinyuan Chen

**Affiliations:** Biomedical & Pharmaceutical Sciences, College of Pharmacy, University of Rhode Island, 7 Greenhouse Road, Pharmacy Building, Room 480, Kingston, RI 02881, USA; yibo_li@uri.edu

**Keywords:** NP, universal T cell vaccine, CpG 1018, CpG, AddaVax, nucleoprotein, influenza

## Abstract

Current influenza vaccines mainly induce neutralizing antibodies against the highly variable surface antigen hemagglutinin and require annual manufacturing and immunization. Different from surface antigens, intracellular nucleoprotein (NP) is highly conserved and has been an attractive target to develop universal T cell vaccines against influenza. Yet, influenza NP protein mainly induces humoral immune responses and lacks the ability to induce potent cytotoxic T lymphocyte (CTL) responses, key for the success of universal T cell vaccines. This study compared CpG 1018 and AddaVax to enhance recombinant NP-induced CTL responses and protection in murine models. CpG 1018 was explored to boost intradermal NP immunization, while AddaVax was explored to boost intramuscular NP immunization due to the high risk of AddaVax adjuvant to induce significant local reactions following intradermal delivery. We found CpG 1018 was highly effective to enhance NP-induced humoral and cellular immune responses superior to AddaVax adjuvant. Furthermore, CpG 1018 potentiated Th1-biased antibody responses, while AddaVax enhanced Th1/Th2-balanced antibody responses. CpG 1018 significantly enhanced IFNγ-secreting Th1 cells, while AddaVax adjuvant significantly increased IL4-secreting Th2 cells. Influenza NP immunization in the presence of CpG 1018 induced significant protection against lethal viral challenges, while influenza NP immunization in the presence of AddaVax failed to elicit significant protection. Our data validated CpG 1018 as an effective adjuvant to enhance influenza NP-induced CTL responses and protection.

## 1. Introduction

Influenza poses a huge threat to global public health and can cause mild to severe respiratory illnesses. Vaccines are regarded as the most effective means to control influenza. Currently approved influenza vaccines mainly induce neutralizing antibodies against viral surface antigen hemagglutinin (HA) [1]. Under immune pressure, surface HA of influenza viruses undergoes frequent mutations [2]. As of such, influenza vaccines are manufactured annually to provide updated protection against potentially new circulating viral strains. Since vaccine viral strains are predicted based on surveillance months before the starting of the flu season, mismatches sometimes occur, resulting in reduced vaccine efficacy [3]. Another disadvantage of current influenza vaccines is that they provide little protection against pandemic viral strains [1].

Different from surface antigens, intracellular antigens, such as nucleoprotein (NP) and matrix protein 1 (M1), are highly conserved and have been attractive targets to develop universal T cell vaccines [4]. Universal T cell vaccines aim to elicit potent cytotoxic T lymphocyte (CTL) responses to eliminate virus-infected cells, reduce disease severity, and promote recovery [4]. Considering externally administered protein antigens are mainly presented on major histocompatibility complex (MHC) II molecules and stimulate antibody responses [5], various strategies have been employed to enable MHC I presentation and CTL induction. These strategies include the employment of DNA/viral vectors to introduce NP or M1 genes into host cells or directly introduce NP or M1 mRNA into host cells [6,7,8,9]. These strategies allow NP or M1 proteins to be expressed intracellularly for presentation on MHC I molecules to stimulate CTL responses. There are also attempts to use virus-like particles (VLPs) to present conserved epitopes of NP or M1 to the immune system [10,11]. This approach takes advantage of VLP’s ability to present its associated antigens to MHC I molecules and stimulate CTL responses. Other approaches include the development of self-assembled nanoparticle vaccines that incorporate conserved CTL epitopes or full-length NP to elicit CTL responses [12,13]. Similar to VLPs, self-assembled nanoparticle vaccines also deliver associated vaccine antigens to MHC I presentation pathway and elicit CTL responses.

At least two universal T cell vaccines against influenza have been tested in clinical trials. Modified vaccinia virus-expressing NP and M1 (MVA − NP + M1) was recently explored in a phase 2b clinical trial [14]. Although MVA − NP + M1 was well tolerated, it failed to reduce laboratory-confirmed influenza when compared to placebo [14]. OVX836 is a recombinant NP-based universal T cell vaccine prepared by self-assembling nanoparticle technology [13]. OVX836 was tested in a phase 2a clinical trial and the 180 µg rather than the 90 µg vaccine dose showed a promising sign to reduce influenza-like illnesses within 14 days of vaccination although a definitive clinical benefit needs to be assessed in future clinical trials [15].

Besides the above strategies, Th1 adjuvants, such as CpG [16], hold a promise to boost protein-based NP or M1 vaccination to elicit CTL responses and protective immunity. CpG adjuvants contain unmethylated CpG motifs and activate toll-like receptor (TLR) 9 with major expression in plasmacytoid dendritic cells (pDCs) and B cells in humans and broader expression in monocytes, macrophages, and conventional DCs in mice [16]. Murine-specific CpG 1826 was explored to boost recombinant NP or NP−M2e fusion protein immunization in murine models [17,18]. In these studies, relatively weak enhancement of protection was observed by incorporation of CpG 1826 adjuvant, likely due to the use of endotoxin-contaminated vaccines (2000 EU/mg for NP−M2e) [17,18]. To explore whether CpG adjuvant has a beneficial effect in boosting recombinant NP (rNP) immunization at low endotoxin levels, we expressed rNP in E. Coli and reduced endotoxin levels to meet the standards of vaccine formulation for preclinical studies (<20 EU/mL) [19]. Furthermore, we used a clinically approved CpG 1018 adjuvant instead of murine specific CpG 1826 considering CpG 1018 has broad effectiveness in rodents, non-human primates, and humans and there is no need to change CpG sequences when vaccines are tested in different animal species or in humans [20]. We found CpG 1018 was highly effective to enhance rNP-induced CTL responses and protection against lethal viral challenges in murine models, while AddaVax adjuvant failed to enhance rNP-induced protection although it significantly increased rNP-induced Th2 and IgG1 antibody responses.

## 2. Materials and Methods

### 2.1. Reagents

CpG 1018 was custom-synthesized by Trilink Biotechnologies (San Diego, CA, USA). AddaVax was obtained from InvivoGen (vac–adx–10, San Diego, CA, USA). Reagents used in molecular cloning, such as Phusion DNA polymerase and restriction enzymes, were purchased from New England Biolabs (NEB, Ipswich, MA, USA). Fluorescence-conjugated antibodies used in immunostaining and flow cytometry were obtained from BioLegend (San Diego, CA, USA). TMB substrate was purchased from Thermo Fisher Scientific (34028, Waltham, MA, USA). Horseradish peroxidase (HRP)-conjugated sheep anti-mouse IgG secondary antibody was purchased from Cytiva (NA931, Marlborough, MA, USA). HRP-conjugated anti-mouse IgG1 was purchased from Invitrogen (046120, Waltham, MA, USA). HRP-conjugated anti-mouse IgG2c was purchased from Bethyl Laboratories (A90–136P, Montgomery, TX, USA). 

### 2.2. Recombinant Plasmid Construction 

NP gene of influenza pandemic 2009 H1N1 virus (A/California/07/2009) with a full length of 1497 bp was obtained from PubMed (GenBank: CY266194.1). Nde I sequence (CATATG, 26–31) within the NP gene was modified to CTTATG to facilitate the construction process. Full gene was synthesized by Thermo Fisher Scientific (Waltham, MA, USA) and amplified with forward primer (5′-GGCGCATATGGCGTCTCAAGGCACCAAACGATCTTATGAACAAATGG-3′, Nde I restriction site underlined) and reverse primer (5′-GGCGCTCGAGACTGTCATACTCCTCTGCATTGTCTCCGAAG-3′, Xho I restriction site underlined). PCR products and pET–29a plasmids were subjected to Nde I and Xho I digestion and then ligation. Successful ligation was confirmed by sequencing. 

### 2.3. Protein Expression and Purification

Recombinant plasmids were transformed into competent BL21 cells. Overnight bacteria were diluted 1:100 into fresh LB medium and grown to OD_600 nm_ (0.8–1.0). Isopropyl-β-D-thiogalactoside (IPTG) was added at a final concentration of 0.1 mM to stimulate protein expression. Bacteria were harvested 3 h later by centrifugation and then lysed in lysis buffer (50 mM Tris–HCl, 300 mM NaCl, pH 8.0) by sonication. After centrifugation, supernatants were loaded onto Ni–NTA column followed by washing and elution with the above lysis buffer supplemented with 0.2 M imidazole. Purified rNP was dialyzed against phosphate–buffered saline (PBS) followed by SDS–PAGE analysis.

### 2.4. Endotoxin Removal

Endotoxin in rNP was removed by Pierce™ High Capacity Endotoxin Removal Resin (88270, Thermo Fisher Scientific, Waltham, MA, USA) following procedures recommended by the manufacturer. Endotoxin levels were measured with a commercial endotoxin quantification kit (A39552, Thermo Fisher Scientific, Waltham, MA, USA).

### 2.5. Immunization

Animal experiments were approved by the Institutional Animal Care and Use Committee of University of Rhode Island (URI) with approval #AN1516–004. Male C57BL/6 mice (6–8 weeks old, Jackson Laboratories) were subjected to hair removal on the lateral back skin one day before experiment as in our previous report [21]. Mice were intradermally injected with 5 μg rNP (endotoxin level < 50 EU per mg of protein) alone or in the presence of 40 μg CpG 1018 [22], or intradermally injected with PBS, or intramuscularly injected with 5 μg rNP admixed with AddaVax (1:1 volume ratio). Injection volume was 20 µl regardless of groups. Thus, the calculated endotoxin level of rNP was <12.5 EU/mL within the recommended endotoxin level of subunit vaccines for preclinical studies (<20 EU/mL) [19]. Immunizations were repeated 3 and 6 weeks later. 

### 2.6. Antibody Titer

Serum anti-NP antibody titer was measured by enzyme-linked immunosorbent assay (ELISA) as in our previous report [23]. Briefly, rNP—coated 96—well ELISA plates were incubated with 4-fold serial dilutions of immune sera. After washing, plates were incubated with HRP-conjugated anti-mouse IgG, IgG1, or IgG2c antibodies. After washing, plates were incubated with TMB substrate. Optical absorbance (OD_450 nm_) was read in a microplate reader (Molecular Devices, San Jose, CA) after adding 1 M H_2_SO_4_.

### 2.7. Cellular Immune Response

NP-specific CD4^+^ and CD8^+^ T cells in peripheral blood mononuclear cells (PBMCs) were quantified as shown in our previous report [23]. Briefly, blood (~50 μL) was collected into heparinized tubes. PBMCs were harvested after red blood cell (RBC) lysis and stimulated with 1 μg/mL rNP in the presence of 4 μg/mL anti-CD28 antibodies overnight. PBMCs were further treated with Brefeldin A (420601, BioLegend, San Diego, CA, USA) to block extracellular cytokine secretion. PBMCs were harvested 5 h later and stained with fluorescence-conjugated antibodies against CD4 (RM4–5) and CD8 (53–6.7). PBMCs were fixed, permeabilized, and then stained with fluorescence-conjugated antibodies against IFNγ (XMG1.2) and IL4 (11B11). PBMCs were then subjected to flow cytometry analysis in BD FACSVerse.

### 2.8. Lethal Viral Challenge

Mice were transferred to the animal biosafety level 2 (ABSL2) facility of URI for challenge. Mice were intranasally instilled with 8× LD50 of mouse-adapted influenza pandemic 2009 H1N1 (pdm09) viruses under light anesthesia as in our previous report [24]. Body weight and survival were monitored daily for 14 days. Mice with body weight loss more than 20% were euthanized (humane endpoint).

### 2.9. Statistics 

Values were expressed as mean ± SEM (standard error of the mean). One-way or two-way analysis of variance (ANOVA) with Tukey’s multiple comparison test was used to compare differences among groups except otherwise specified. *p*-value was calculated by PRISM software and considered significant if it was less than 0.05.

## 3. Results

### 3.1. Construction, Expression, and Purification of rNP

**Figure 1:**
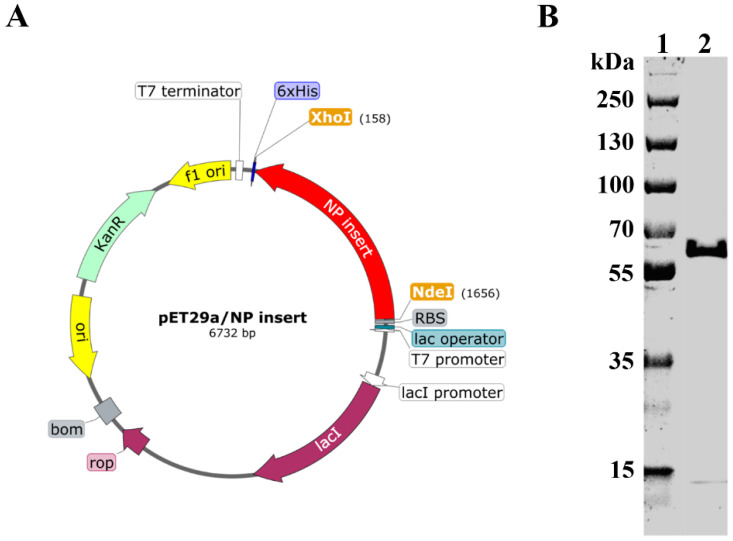
Construction and expression of rNP. (**A**). Schematic illustration of recombinant plasmid construction (prepared by SnapGene). (**B**). Recombinant NP was expressed, purified, and subject to SDS–PAGE analysis. Lane 1: Marker. Lane 2: rNP.

Recombinant plasmid was prepared by insertion of NP gene between Nde I and Xho I sites of the pET−29a vector (Figure 1A). Recombinant plasmid was then transformed into BL21 cells. Recombinant NP (rNP) was expressed, purified, and subjected to SDS−PAGE analysis. As shown in Figure 1B, rNP showed a good purity at the expected theoretical molecular weight (57.1 kDa). 

### 3.2. CpG 1018 Enhances rNP-Induced Antibody Responses

**Figure 2:**
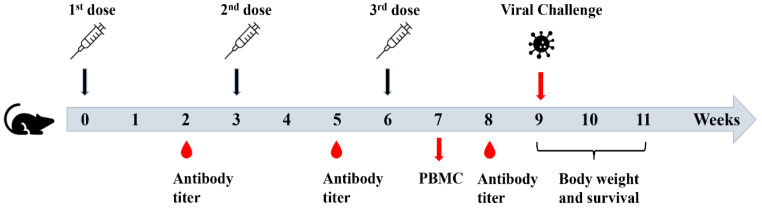
Schematic illustration of experimental procedures.

Recombinant NP is expected to mainly induce antibody responses with a weak ability to stimulate CTL responses, critical for development of effective T cell vaccines. Incorporation of Th1 adjuvants into rNP is promising to enhance the stimulation of rNP-specific CTL responses. FDA—approved Th1 adjuvant (CpG 1018) and more balanced Th1/Th2 adjuvant (MF59—mimetic AddaVax) were explored to enhance rNP-induced humoral and cellular immune responses and protection against lethal viral challenges in murine models. Mice were subjected to intradermal (ID) immunization of rNP alone or in the presence of CpG 1018, intramuscular (IM) immunization of rNP in the presence of AddaVax, or intradermally injected with PBS. The ID route was chosen to deliver rNP in the presence of CpG 1018 due to its promise to induce more potent immune responses than the IM route and the good safety of CpG adjuvant for ID delivery [25,26]. In contrast, AddaVax adjuvant has a high risk to induce significant local reactions following ID delivery [27]. As of such, the IM route was chosen to deliver rNP in the presence of AddaVax adjuvant. Immunization was repeated every three weeks for a total of three times (Figure 2). Serum anti-rNP antibody titer was measured two weeks after each immunization.

**Figure 3:**
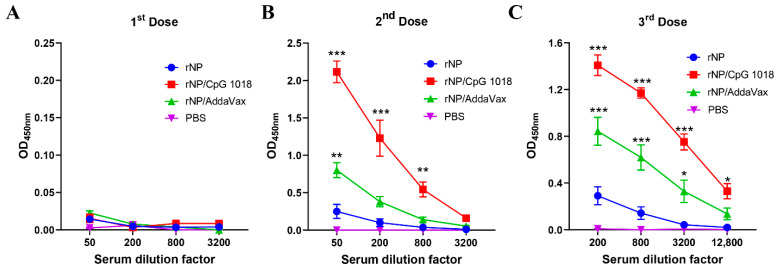
CpG 1018 more significantly enhances rNP-induced antibody responses than AddaVax. Mice were subjected to ID rNP immunization alone or in the presence of CpG 1018 adjuvant or IM rNP immunization in the presence of AddaVax, or intradermally injected with PBS. Immunization was repeated every three weeks for total three times. rNP dose remained the same (5 µg) among groups except the PBS group. Serum anti-rNP antibody titer was measured two weeks after each immunization and shown in (**A**–**C**), respectively. Data were expressed as mean ± SEM. *n* = 4/group. Two-way ANOVA with Turkey’s multiple comparison test was used to compare differences of OD_450 nm_ between adjuvant and rNP alone groups. *, *p* < 0.05; **, *p* < 0.01; ***, *p* < 0.001. Experiments were repeated once with similar results.

One dose of immunization, regardless of group, elicited weak antibody responses (Figure 3A). Significant anti-NP antibody responses were elicited after a second dose (Figure 3B). ID rNP immunization in the presence of CpG 1018 elicited the most potent anti-NP antibody responses, followed by IM rNP immunization in the presence of AddaVax, and then ID rNP immunization alone (Figure 3B). Furthermore, ID rNP immunization in the presence of CpG 1018 elicited significantly higher anti-NP antibody responses than IM rNP immunization in the presence of AddaVax after the second and third doses (Table 1). 

**Figure 4:**
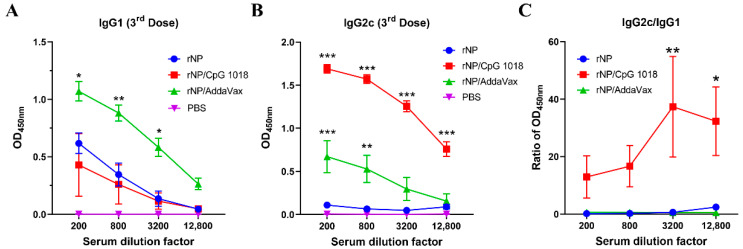
CpG 1018 induces Th1-biased antibody responses. (**A**,**B**). Serum anti-NP IgG1 and IgG2c antibody responses after the third dose were measured and shown in (**A**,**B**), respectively. (**C**). Ratio of anti-NP IgG2c/IgG1 antibody responses. Due to the extremely low absorbance values of PBS group in A and B, the ratio of IgG2c/IgG1 in this group was not accurate and thus not shown in (**C**). Data were expressed as mean ± SEM. *n* = 4/group. Two-way ANOVA with Turkey’s multiple comparison test was used to compare differences between adjuvant and rNP alone groups. *, *p* < 0.05; **, *p* < 0.01; ***, *p* < 0.001. Experiments were repeated once with similar results.

Anti-NP IgG1 and IgG2c antibody responses were also measured after the third dose. As shown in Figure 4A, rNP alone induced weak IgG1 antibody responses. AddaVax but not CpG 1018 adjuvant significantly increased anti-NP IgG1 antibody production (Figure 4A). As expected, rNP alone failed to induce significant anti-NP IgG2c antibody responses (Figure 4B). Both AddaVax and CpG 1018 increased anti-NP IgG2c antibody responses while CpG 1018 adjuvant more vigorously enhanced anti-NP IgG2c antibody production (Figure 4B). The ratio of IgG2c/IgG1 antibody responses was significantly increased at the two highest dilution factors by incorporation of CpG 1018 adjuvant (Figure 4C), hinting the induction of Th1-biased antibody responses. The ratio of IgG2c/IgG1 antibody responses showed no significant change after incorporation of AddaVax adjuvant, hinting the induction of balanced Th1/Th2 antibody responses. ID rNP immunization in the presence of CpG induced no visible local reactions and caused no significant increase in rectal temperature, hinting good local and systemic safety of such immunization. 

### 3.3. CpG 1018 Enhances rNP-Induced Cellular Immune Responses

**Figure 5:**
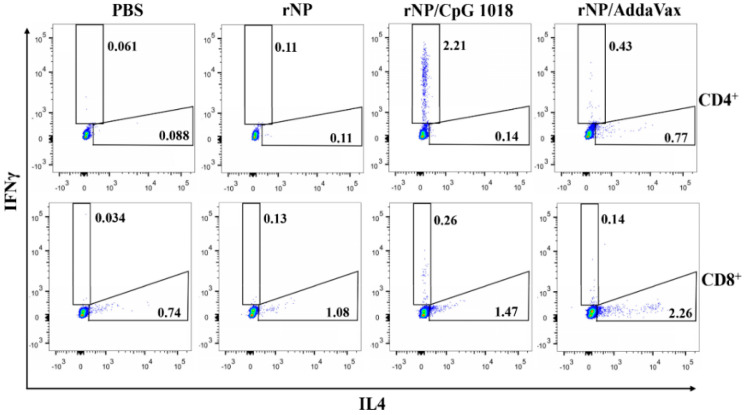
CpG 1018 induces significant IFNγ-secreting CD4^+^ and CD8^+^ T cells. PBMCs of the diverse rNP- or PBS-immunized mice were collected one week after the third dose and stimulated with rNP followed by intracellular cytokine staining and flow cytometry analysis. Cells were first gated based on FSA and SSC and then based on CD4 and CD8 expression. IFNγ- and IL4-secreting cells in CD4^+^ and CD8^+^ T cells were then gated and representative dot plots were shown. Experiments were repeated once with similar results.

NP-specific cellular immune responses were also evaluated. Briefly, one week after the third dose, PBMCs were collected and stimulated with rNP overnight. The percentage of intracellular IFNγ- and IL4-secreting cells in CD4^+^ and CD8^+^ T cells were compared among groups. As shown in Figure 5, ID rNP immunization in the presence of CpG 1018 stimulated significant IFNγ-secreting CD4^+^ T cells or Th1 cells and failed to elicit significant IL4-secreting Th2 cells, in line with the induction of Th1-biased antibody responses in the above studies. In contrast, IM rNP immunization in the presence of AddaVax increased both IFNγ- and IL4-secreting CD4^+^ T cells (Figure 5), hinting induction of balanced Th1/Th2 immune responses. As for CD8^+^ T cells, IM rNP immunization in the presence of AddaVax stimulated IL4-secreting CD8^+^ T cells, while ID rNP immunization in the presence of CpG 1018 stimulated IFNγ-secreting CD8^+^ T cells (Figure 5). 

**Figure 6:**
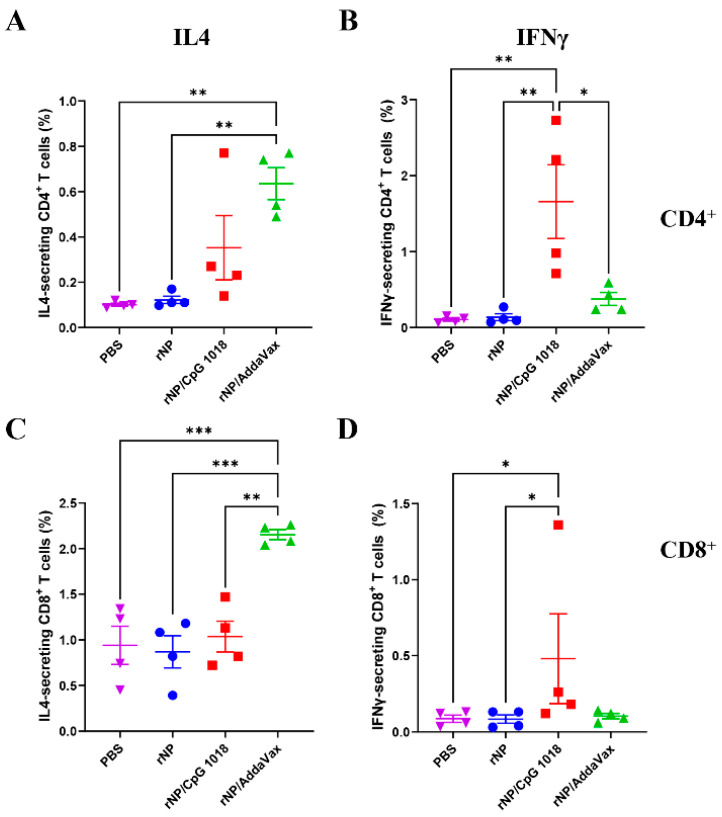
Comparison of rNP-specific cellular immune responses in PBMCs. IL4- and IFNγ-secreting CD4^+^ and CD8^+^ T cells in PBMCs (stained and analyzed in Figure 5) were compared among groups. (**A**). Percentage of IL4-secreting cells in CD4^+^ T cells. (**B**). Percentage of IFNγ-secreting cells in CD4^+^ T cells. (**C**). Percentage of IL4-secreting cells in CD8^+^ T cells. (**D**). Percentage of IFNγ-secreting cells in CD8^+^ T cells. *n* = 4. One-way ANOVA with Turkey’s multiple comparison test was used to compare differences between groups in (**A**–**C**). Kruskal–Wallis test with Dunn’s multiple comparisons was used to compare differences between groups in (**D**). *, *p* < 0.05; **, *p* < 0.01; ***, *p* < 0.001. Experiments were repeated once with similar results.

The frequencies of IL4- and IFNγ-secreting CD4^+^ and CD8^+^ T cells were then compared. As shown in Figure 6, ID rNP in the presence of CpG 1018 induced significantly higher percentages of IFNγ-secreting CD4^+^ and CD8^+^ T cells as compared to ID rNP immunization alone. In comparison, IM rNP immunization in the presence of AddaVax induced significantly higher percentages of IL4-secreting CD4^+^ and CD8^+^ T cells as compared to ID rNP immunization alone or ID rNP immunization in the presence of CpG 1018 (Figure 6). ID rNP immunization alone failed to significantly increase IFNγ- or IL4-secreting CD4^+^ or CD8^+^ T cells when compared to PBS injection (Figure 6).

### 3.4. CpG 1018 Increases rNP-Induced Protection against Lethal Viral Challenges

**Figure 7:**
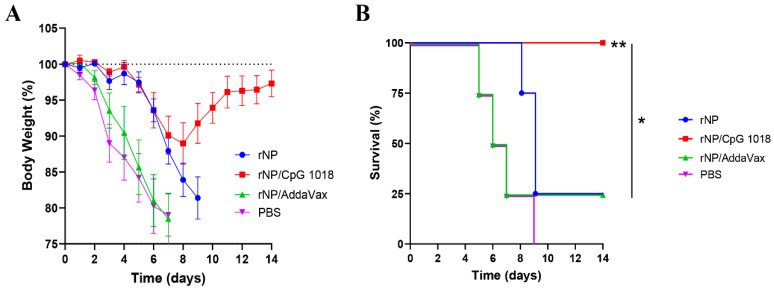
CpG 1018 enhances rNP-induced protection in challenge studies. Mice were challenged 3 weeks after the last immunization. Body weight loss (**A**) and survival (**B**) were monitored daily for 14 days. Mice were humanely euthanized when the body weight loss reached 20% threshold. *n* = 4. Log-rank test with Bonferroni correction was used to compare differences between groups in (**B**). *, *p* < 0.05; **, *p* < 0.01. Experiments were repeated once with similar results.

Mice were challenged with a lethal dose of mouse-adapted influenza pandemic 2009 H1N1 viruses three weeks after the third immunization to evaluate the protection. As shown in Figure 7A, all groups showed a similar trend of body weight loss in the first few days after challenge except the body weight loss was delayed by a few days in rNP and rNP/CpG 1018 groups. Mice in rNP/CpG 1018 group lost a maximum of 11% body weight on day 8, while mice in other groups lost ~20% body weight in 7–9 days. Mice in rNP/CpG 1018 group started to recover after day 8, while the majority of mice in other groups either died or reached humane euthanasia endpoint after day 8. All mice survived in rNP/CpG 1018 group, while only 25% mice survived in rNP or rNP/AddaVax group and all mice died in PBS group (Figure 7B). 

## 4. Discussion

Our study indicated CpG 1018 was highly effective to enhance rNP-induced CTL responses and protection in murine models. In contrast, MF59-mimetic AddaVax adjuvant failed to significantly enhance rNP-induced CTL responses or protection in murine models. Rather than inducing IFNγ-secreting CD8^+^ T cells, rNP immunization in the presence of AddaVax adjuvant induced potent IL4-secreting CD8^+^ T cell responses. Although the function of IL4-secreting CD8^+^ T cells remained to be explored, the lack of significant protection in rNP/AddaVax group hinted the weak cytotoxicity of IL4-secreting CD8^+^ T cells. In agreement, IL4-secreting CD8^+^ T cells were found to have diminished perforin/granzyme-mediated cytotoxicity and could even dampen the anti-viral ability of CD8^+^ T cells [28]. The strong induction of anti-NP CTL responses in rNP/CpG 1018 group was likely due to the induction of cross-presentation by CpG adjuvant [29]. 

CpG 1018 adjuvant also vigorously enhanced rNP-induced humoral immune responses. Serum anti-NP antibody titer was significantly higher in rNP/CpG 1018 group than that in rNP/AddaVax group. Interestingly, rNP immunization with CpG 1018 adjuvant exclusively enhanced anti-NP IgG2c but not IgG1 antibody production, while rNP immunization with AddaVax adjuvant enhanced both IgG2c and IgG1 antibody production. This result hints CpG 1018 potentiates Th1-biased antibody responses, while AddaVax potentiates more balanced Th1/Th2 antibody responses. In line with this result, CpG 1018 adjuvant increased IFNγ-secreting CD4^+^ T cells or Th1 cells but not IL4-secreting CD4^+^ T cells or Th2 cells, while AddaVax adjuvant increased both though only Th2 cells showed a statistically significant increase. 

Although the different humoral, helper T cell, and CTL responses between the two adjuvant groups may be caused by the diverse immunization routes, adjuvants likely play a dominant role in shaping vaccine-induced immune responses (Th1/Th2 and antibodies/CTLs). A literature search found only a few studies directly compared the quality of immune responses after different routes of vaccine delivery. One study found ID and IM delivery of OVA-loaded nanoparticles elicited a similar IgG2a titer, while intralymphatic injection induced the strongest IgG2a titer and subcutaneous injection induced the weakest IgG2a titer [30]. The same study found IgG1 titer was less sensitive to the change of immunization routes [30]. Another study found polyfunctional CD8^+^ T cells could be more preferentially induced after ID or IM delivery of modified vaccinia virus Ankara vaccine, while subcutaneous route could induce higher titers of neutralizing antibody responses [31]. These studies hinted ID and IM deliveries likely induced similar types of immune responses although direct comparison of CpG 1018 and AddaVax in the same route of rNP immunization is required to exclude the potential impact of immunization routes on the observed differential immune responses. 

Influenza NP in the presence of CpG 1018 conferred significant protection against homologous viruses. A similar trend of initial body weight loss in all groups indicated NP-based vaccines conferred protection at a later stage of infection due to the lack of neutralizing antibodies. Mice of rNP/CpG 1018 group lost a maximum of 11% body weight, while most of the mice in other groups lost a maximum of more than 20% body weight. Mice of rNP/CpG 1018 group started to recover after day 8 of infection and recovered more than 97% body weight on day 14. All mice survived in rNP/CpG 1018 group, while at least 75% mice died in other groups. The survival rate was significantly higher in rNP/CpG 1018 group than that in other groups. Two prior studies explored the potency of murine-specific CpG 1826 adjuvant to boost recombinant NP and NP-M2e immunization [17,18]. It was found that CpG 1826 alone could not significantly enhance the protection [17,18]. The underlying reason remained to be explored and might be related to the use of endotoxin-contaminated NP and NP-M2e [17,18]. It also remained to be explored whether the different immunization routes and CpG adjuvants used between our study and the prior two studies caused the discrepancy. To our knowledge, this might represent the first study to show the high effectiveness of clinical CpG 1018 adjuvant to boost ID rNP immunization to induce potent CTL responses and protection against lethal viral challenges. Our study encourages evaluation of CpG 1018 to enhance rNP-induced CTL responses and protection against heterologous and heterosubtypic viruses. 

We believe the protection observed in rNP/CpG 1018 group was mainly contributed by IFNγ-secreting CD8^+^ T cells. One study found that non-neutralizing anti-NP antibodies also showed protective efficacy [32]. The relative contribution of anti-NP antibodies to the overall protection in our study is expected to be very limited in consideration of the little or no protection observed in rNP/AddaVax group, which elicited significantly higher anti-NP antibody titer than rNP immunization alone. Our study supports CpG 1018 to be a potentially better adjuvant than AddaVax for development of an influenza NP-based universal T cell vaccine. Our current study only used NP to test the feasibility of CpG 1018 adjuvant to enhance the protective efficacy. Future studies can combine NP with M1 or other conserved universal influenza vaccine antigens, such as M2e, to expand the breadths of protection in the presence of CpG 1018 adjuvant. Different from other strategies in universal T cell vaccine development, such as DNA/viral/mRNA vaccines and particulate delivery platforms, CpG 1018-adjuvanted rNP does not involve complex design and manufacturing and represents a traditional vaccine type that is expected to be well accepted by the general public. 

## Figures and Tables

**Table 1:** Comparison of anti-NP antibody responses between the two adjuvant groups.

	1:50	1:200	1:800	1:3200	1:12,800
2nd dose	*p* < 0.001	*p* < 0.001	*p* < 0.05	NS	-
3rd dose	-	*p* < 0.001	*p* < 0.001	*p* < 0.001	NS

## Data Availability

The data presented in this study are available on request from the corresponding author.
